# Evaluating the efficacy of smoke management technologies in laparoscopic sleeve gastrectomy: insights from a prospective, single-centre comparative study

**DOI:** 10.1038/s41598-026-43227-y

**Published:** 2026-03-23

**Authors:** Cédric R. D. Demtröder, Daniel Göhler, Kathrin Oelschlägel, Claudia Jahn-Wolf, Hülya Agarius, Peter Kirchmeyer, Fabian Kockelmann, Sébastien Roger, Mehdi Ouaissi, Dmitrij Dajchin, Urs Giger-Pabst

**Affiliations:** 1 Department of Surgery, Martinus-Krankenhaus, Düsseldorf, Germany; 2 Topas GmbH, Dresden, Germany; 3 Research Group Mechanical Process Engineering, Institute of Process Engineering and Environmental Technology, Technische Universität Dresden, Dresden, Germany; 4 Department of Surgery, Klinikum Dortmund, Hospital of the Witten/Herdecke University, Dortmund, Germany; 5 UMR1327 ISCHEMIA “Membrane Signalling and Inflammation in Reperfusion Injuries”, Université de Tours, Tours, France; 6 Department of Digestive, Oncological, Endocrine, Hepato-Biliary, Pancreatic and Liver Transplant Surgery, University Hospital of Tours, Tours, France; 7 Fliedner Fachhochschule, University of Applied Sciences Düsseldorf, Düsseldorf, Germany

**Keywords:** Laparoscopy, Gastric sleeve, Surgical smoke management technologies, Smoke evacuation, Electrostatic aerosol precipitation, Diseases, Medical research

## Abstract

Surgical power devices generate surgical smoke that may contain infectious components. Various technologies have been developed to improve surgical smoke management, but comparative performance data from human studies are limited. A prospective, single-centre study was performed for evaluating three smoke management technologies - continuous passive filtration (CPF), electrostatic precipitation (ESP), and continuous active filtration (CAF) - during laparoscopic sleeve gastrectomy in 15 bariatric patients. Surgical smoke concentration was monitored by condensation particle counting and single particle light scattering. Efficiency of intraoperative smoke clearance was assessed by the concentration half-life (T_1/2_). Secondary outcomes included total CO_2_ consumption, intraoperative pressure stability, and intraoperative visibility. ESP showed the highest smoke clearance efficiency (T_1/2_ = 7.2 s), followed by CAF (18.3 s) and CPF (20.6 s) with significant differences. Total CO_2_ consumption was highest for CAF (452.0 L) compared CPF (242.0 L) and ESP (80.1 L). All groups maintained a stable capnoperitoneal pressure and a good/very good intraoperative visibility. Electrostatic precipitation showed the lowest CO_2_ consumption and significantly higher smoke particle removal efficiency compared to continuous active/passive filtration. All technologies provided good/very good intraoperative visibility and capnoperitoneal pressure stability.

**Supplementary Information:**

The online version contains supplementary material available at 10.1038/s41598-026-43227-y.

## Introduction

Modern surgery is closely linked to the advent of surgical power devices (SPDs), which have improved dissection and hemostasis, reduced operating times, made procedures safer and shortened hospital stays compared to classical surgical instruments^1,2^. Unfortunately, the use of SPDs is accompanied by the formation of toxic/mutagenic surgical smoke due to combustion, vaporization, coagulation, and mechanical disruption of tissue and body fluids^3^. Chemical composition, particle size, and amount of surgical smoke depend on the type of operated SPD, the amount of applied energy, and the processed tissue^4,5^. Some compounds of surgical smoke may exceed established exposure limits^6–10^. A further concern is that surgical smoke can contain next to water vapor, toxic gases and non-viable particles also viable malignant cells, bacteria and viruses like human papillomavirus and human immunodeficiency virus^11–14^.

The relevance of the health risk by surgical smoke is reflected in data from US health authorities, which indicate that more than 500,000 US healthcare workers are regularly exposed^3^. To improve intraperitoneal clearance during laparoscopic and robotic surgeries, surgical smoke is often released intentionally into the operating room^15^. Unfortunately, general operating room ventilation is not sufficient to eliminate surgical smoke^16^. Wearing of conventional masks^17^ or masks with higher filtration efficiency like N95/FFP2 or N99/FFP3^18^ can significantly reduce exposure, but cannot provide absolute safety. Thus, there is broad consensus that the safe management of surgical smoke, particularly in laparoscopic surgery, requires the rigorous use of smoke management technologies^19^. Existing smoke management technologies for laparoscopic surgery based on continuous passive filtration (CPF), continuous active filtration (CAF) or electrostatic precipitation (ESP)^20,21^.

With the onset of the COVID-19 pandemic, concerns about aerosolisation of virus-containing particles and their potential release from the capnoperitoneum into the operating room led to the suggestion that laparoscopic surgery should be replaced by open surgical interventions if patients are infected by SARS-CoV-2 or if their status is unknown^22^. The lack of data on the efficacy of common smoke management technologies initiated the first comparative preclinical ex-vivo^23^ and in-vivo animal^24^ studies. In these studies, different laparoscopic procedures (i.e., cholecystectomy, atypic liver resection, colon surgery) were performed in order to identify appropriate comparative parameter (like the half-life of particle number concentration or the coefficient of variation of the capnoperitoneal pressure) taking into account both smoke elimination efficiency and freedom of surgery, and rank common smoke management technologies according to their performance.

To verify the ex-vivo and in-animal preclinical findings concerning the performance of surgical smoke management technologies^23,24^ under most realistic conditions, the present clinical trial was conducted in patients undergoing laparoscopic gastric sleeve. To be consistent with^24^, identical parameters, such as the half-life of particle number concentration for evaluating surgical smoke removal efficiency and the coefficient of variation of capnoperitoneal pressure for assessing capnoperitoneal stability, were adopted from this study. The three technologies reported in^24^ were selected because they demonstrated the highest effectiveness and practical applicability during laparoscopic surgery.

## Materials and methods

### Legal background

Prospective study at the Martinus Hospital, Düsseldorf, Germany, a centre of excellence for bariatric and metabolic surgery. The study was conducted in accordance with the guidelines of the Declaration of Helsinki, and each patient gave oral and written informed consent. Approval was obtained from the Ethics Committee of the Medical Chamber North Rheine, Düsseldorf, Germany (file #2022361). German Clinical Trials Register (file #DRKS00030869). The study was registered on 14 December 2022 prior to the start of the clinical trial^25,26^. There were no changes or deviations from the study protocol during or after the end of the trial.

### Patient selection and study group assignment

Laparoscopic gastric sleeve^27^ is a frequently performed and highly standardised surgical intervention and was therefore chosen for this trial. Patients were selected based on national guidelines^28^. The only restrictions were an age of at least 18 years and the absence of previous metabolic surgery. The study was conducted on three consecutive days in the same operating theatre and patients were randomly allocated to one of three study groups (A, B, C) independently of the blinded coordination manager and the surgical team. All procedures were performed by the same team of physicians and scrub nurses, with standardised surgical procedures and technical equipment, in accordance with the study protocol. Surgical smoke particle concentration measurements were conducted in the capnoperitoneum, as the data obtained are more valid compared to extraperitoneal measurements and are therefore also representative of the risk of extraperitoneal smoke exposure^24^.

The primary endpoint of this study was the efficiency of intraoperative smoke removal, assessed by the concentration half-life (T_1/2_). Accordingly, also the required sample size was calculated based on T_1/2_ values from translational-relevant preclinical data of large animal in-vivo experiments^24^. A minimum sample size of 1 to 3 patients was calculated using the pooled standard deviation, effect size and a two-sided t-test with α = 0.05 and 80 % power. To ensure robust differentiability, 5 patients were used per study group. Secondary outcomes included total CO_2_ consumption, intraoperative pressure stability, visual quality, and operating time.

### Operative setup, volumetry of capnoperitoneum and laparoscopic gastric sleeve

Access to the abdominal cavity was obtained in the left upper quadrant by video-assisted (IMAGE1 S 4U RUBINA, OPAL1 NIR/ICG, Karl Storz, Germany) insertion of a 12 mm trocar Kii Optical Access System (T1). All trocars, except the optic trocar in group C, were from Applied Medical, CA-USA. In study groups A and B, the insufflator (Endoflator UI 500, Karl Storz, Germany) and in group C the continuous pressure insufflator (LEXION AP 50/30, Dach Medical Group GmbH, Austria) were connected to T1. During establishment of a capnoperitoneum of 15 mmHg, the intra-abdominal volume was determined for (5, 10, 15) mmHg. Under video-guidance, insertion of a 12 mm trocar in the midline supraumbilical (T2) and in the right upper abdomen (T3). A 15 mm trocar (T5) was placed subxyphoidal and a 5 mm trocar (T4) in the lateral left upper abdomen. In group C, the CO_2_ inflow was connected to T2 (12 mm, Insuflow®Port, Dach Medical Group GmbH, Austria) and the outflow to T5 (PneuVIEW®XE, Dach Medical Group GmbH, Austria). In Group A and B, the heated CO_2_ insufflation tube was connected to the side valve of T2. Insertion of a 10 mm retractor in T5 to elevate the left liver lobe. Continuous capnoperitoneal pressure monitoring by a precision pressure probe (Almemo 2590-4AS logger with FDA612SR sensor, Ahlborn, Germany) connected to the open side valve of T4. Tissue dissection with an ultrasonic cutter (Sonicision™ 7 Curved Jaw, Medtronic plc, Ireland). For further details on the surgical procedure, refer to existing literature^27^.

### Study groups and smoke management technologies details

**Fig. 1:**
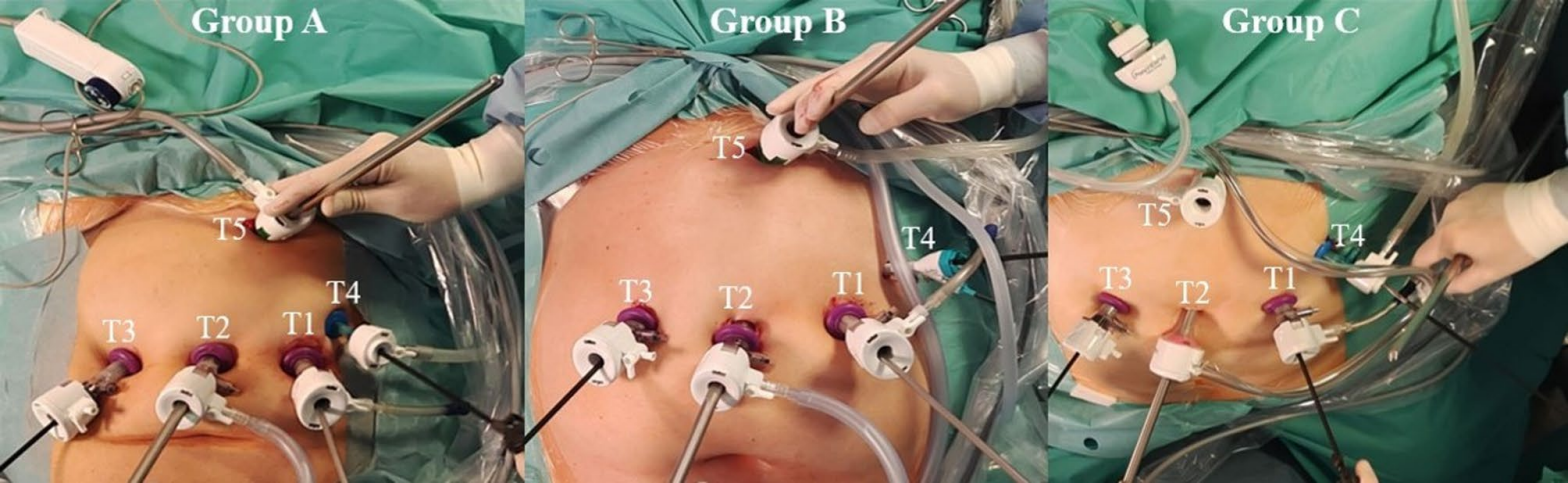
Operative setup and implementation of examined surgical smoke management technologies. Group A: T1 = aerosol sampling; T2 = CO_2_ inflow; T4 = monitoring of capnoperitoneal pressure; T5 = continuous passive filtration (CPF); Group B: T1 = aerosol sampling; T2 = CO_2_ inflow; T4 = trocar for electrostatic smoke precipitation (ESP); T5 = monitoring of capnoperitoneal pressure; Group C (continuous active filtration): T1 = aerosol sampling; T2 = CO_2_ inflow; T4 = monitoring of capnoperitoneal pressure; T5 = CO_2_ outflow.

Intraoperative smoke management technologies were studied in three groups (A, B, C) with 5 patients each. The operational setups are shown in (Fig. 1).

- **Group A:** continuous passive filtration (CPF) with integrated moisture capture system (Valleylab™ Laparoscopic Smoke Evacuation System, Medtronic plc, Ireland) at maximum flow connected to T5.
- **Group B:** continuous electrostatic smoke precipitation (ESP) using the Ultravision™ generator with 5 mm Trocar accessory Ionwand ™ (Alesi Surgical Ltd., UK) at T4.
- **Group C:** continuous active filtration (CAF) with continuous pressure insufflator and integrated smoke evacuation (LEXION AP 50/30) in bariatric mode (40 L/min) with inflow at T2 (Insuflow®Port) and outflow at T5 (PneuVIEW®XE).

### Aerosol-analytical setup for surgical smoke characterisation

**Fig. 2:**
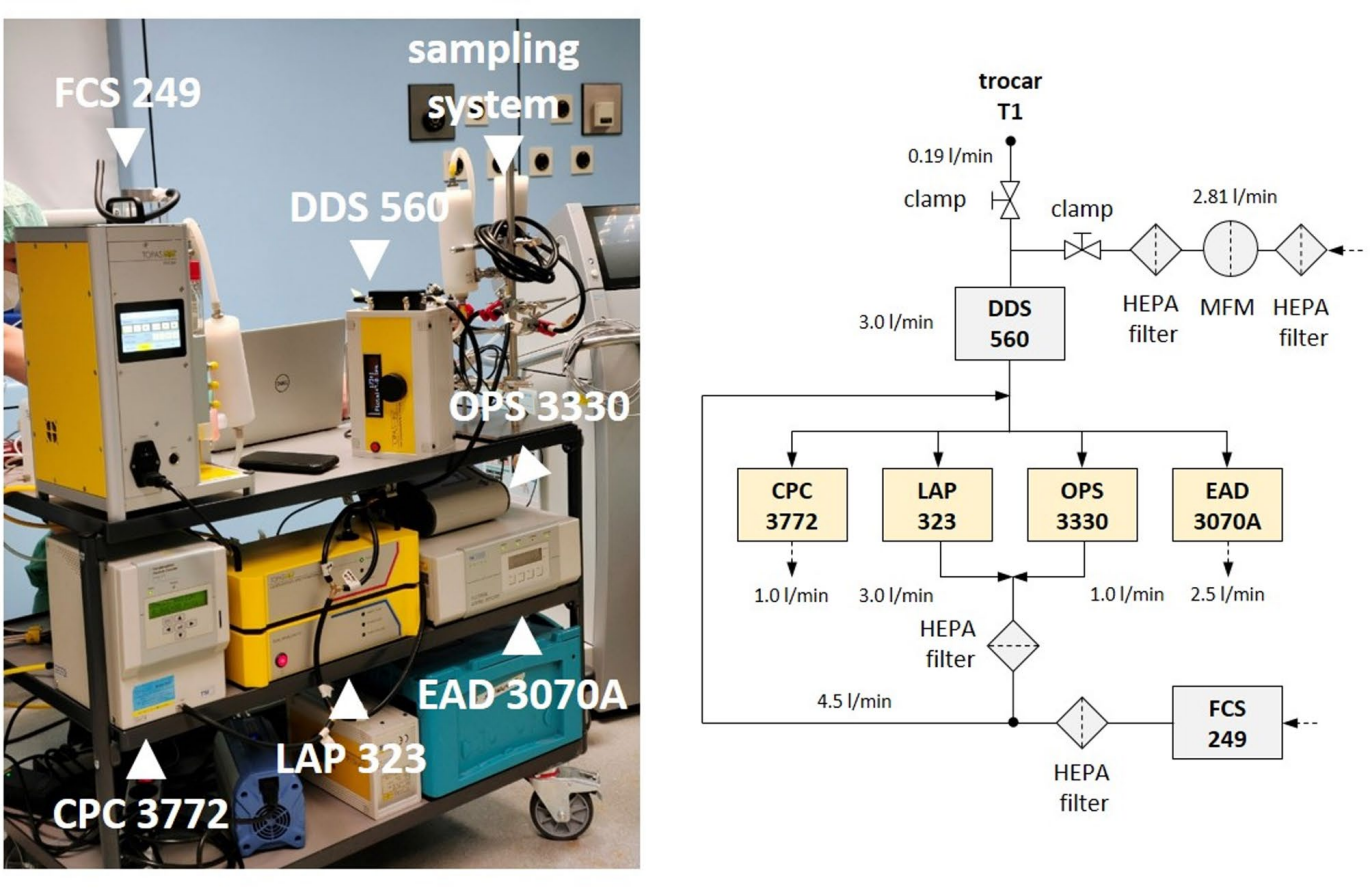
Photographic image (left) and schematic diagram of the operated aerosol-analytical setup (right). *CPC* condensation particle counter, *DDS* dynamic dilution system, *FCS* field calibration system, *LAP* laser aerosol particle size spectrometer, *OPS* optical particle sizer.

The operated aerosol analytical setup (Fig. 2) corresponds widely to the intra-abdominal section of a previous setup^24^.

Aerosol sampling from the side valve of T1 via a 1.5 m electrical-conductive hose line enveloped by a sterile tube cover (Flexasoft®, CADITEC Medical + Technical GmbH, Germany). For proper instrument operation, the sample flow rate of (0.19 ± 0.04) L/min was i) mixed first with 2.81 L/min of particle-free air for air-like conditions and ii) diluted by an aerosol dilution system (DDS 560, Topas GmbH, Germany) and by particle-free air supplied from instrument exhaust and from a flow-controlled aerosol generator (FCS 249, Topas GmbH, Germany) in idle mode. The overall aerosol dilution factor varied between 29 and 654.

Monitoring of particle number concentration was realised by three aerosol-analytical instruments. A condensation particle counter (CPC 3772, TSI Inc., USA) was used for concentrations in the size range of (0.01–10) µm^29^. One (LAP 323, Topas GmbH, Germany) of the two single particle light aerosol spectrometers^30^ monitored concentrations in the size range of (0.15–5) µm, while the other one (OPS 3330, TSI Inc., USA) in the size range of (0.3–10) µm.

### Assessment of intraoperative vision

Intraoperative vision at different stages of the surgical intervention was rated by the surgical team (n = 4) using a 5-point Likert scale^31^. In addition, the number of camera cleaning procedures were protocolled.

### Statistical analyses

Data are presented as median values with standard deviation and as boxplots (median values with interquartile range (IQR) = Q3–Q1). Non-parametric variables were analysed with the Kruskal–Wallis test^32^. Significant differences were assessed with the Mann–Whitney test^33,34^. A value of p < 0.05 represents for each of the mentioned tests a significant difference.

## Results

### Patient characteristics, details about surgery and postoperative outcome

In total, 15 patients with a male-to-female ratio of 1:4 were treated. The overall median age, body weight, and body mass index (BMI) were (34 ± 15.4) a, (125 ± 29.6) kg, and (45.7 ± 6.8) kg/m^2^. There were no significant differences between the groups concerning age and body weight, while next to the sex also significant differences for the BMI between group A and C existed (p_A/C_ = 0.024).

The three smoke management technologies were operated intraoperatively without complications. The median duration of surgery was (46 ± 11) min. The significantly higher operative time of Group C compared to Group A was caused by one patient, who required additional time of approx. 25 min for adhesiolysis of extensive adhesions between the greater omentum and the liver. The postoperative courses were uneventful and all patients were discharged at home on the third postoperative day (i.e., minimum required hospital stay time for sleeve gastrectomy as mandated by the German healthcare system based on diagnosis-related groups^35^; hospital stay time can significantly vary between the different national health care systems).

After one year of follow-up, the median total and excess weight loss were (27.2 ± 8.5) % and (66.1 ± 18.1) %, respectively. There was no significant difference between the three study groups. Table 1 summarises the perioperative and 12-month follow-up data.

**Table 1:** Patient characteristics, details about surgery and postoperative outcome.

property	Unit	Group A	Group B	Group C	Total	*p*_*A/B*_	p_A/C_	*p*_*B/C*_
Smoke management technology	-	CPF	ESP	CAF	-	-	-	-
Number of patients (male/female)	-	5 (0/5)	5 (0/5)	5 (3/2)	15 (3/12)	-	-	-
Age of patient	year	31 ± 18.4	28 ± 5.1	42 ± 18.3	34 ± 15.4	0.174	0.377	0.087
Body weight, preoperative	kg	116 ± 15.0	125 ± 8.0	170 ± 39.5	125 ± 29.6	0.174	0.059	0.174
Body mass index, preoperative	kg/m^2^	43.7 ± 5.5	45 ± 1.4	50.1 ± 8.3	45.7 ± 6.8	0.174	0.024	0.059
Duration of surgery	min	43 ± 9.2	46 ± 2.7	53 ± 15.4	46 ± 11.3	0.087	0.024	0.001
Length of hospital stay	day	3 ± 0	3 ± 0	3 ± 0	3 ± 0	1.000	1.000	1.000
Body weight, postoperative (1 year)	kg	79 ± 7.4	86 ± 7.1	105 ± 31.7	86 ± 25.3	0.232	0.014	0.038
Excess body weight loss, postoperative (1 year)	%	67.7 ± 10.7	71.9 ± 9.7	43.6 ± 20.7	66.1 ± 18.1	0.377	0.038	0.059

Median values ± standard deviation.

*CPF* continuous passive filtration, *CAF* continuous active filtration, *ESP* electrostatic precipitation.

### Capnoperitoneal volume, carbon dioxide consumption and capnoperitoneal stability

A cross-group comparison of the determined capnoperitoneal volumes revealed no statistically significant difference; however, significant variations were observed in dependence of the capnoperitoneal pressure. The mean capnoperitoneal volume was determined to be (0.6 ± 0.8) L for 5 mmHg, (3.7 ± 1.5) L for 10 mmHg and (6.0 ± 1.7) L for 15 mmHg (see Fig. S1).

**Fig. 3:**
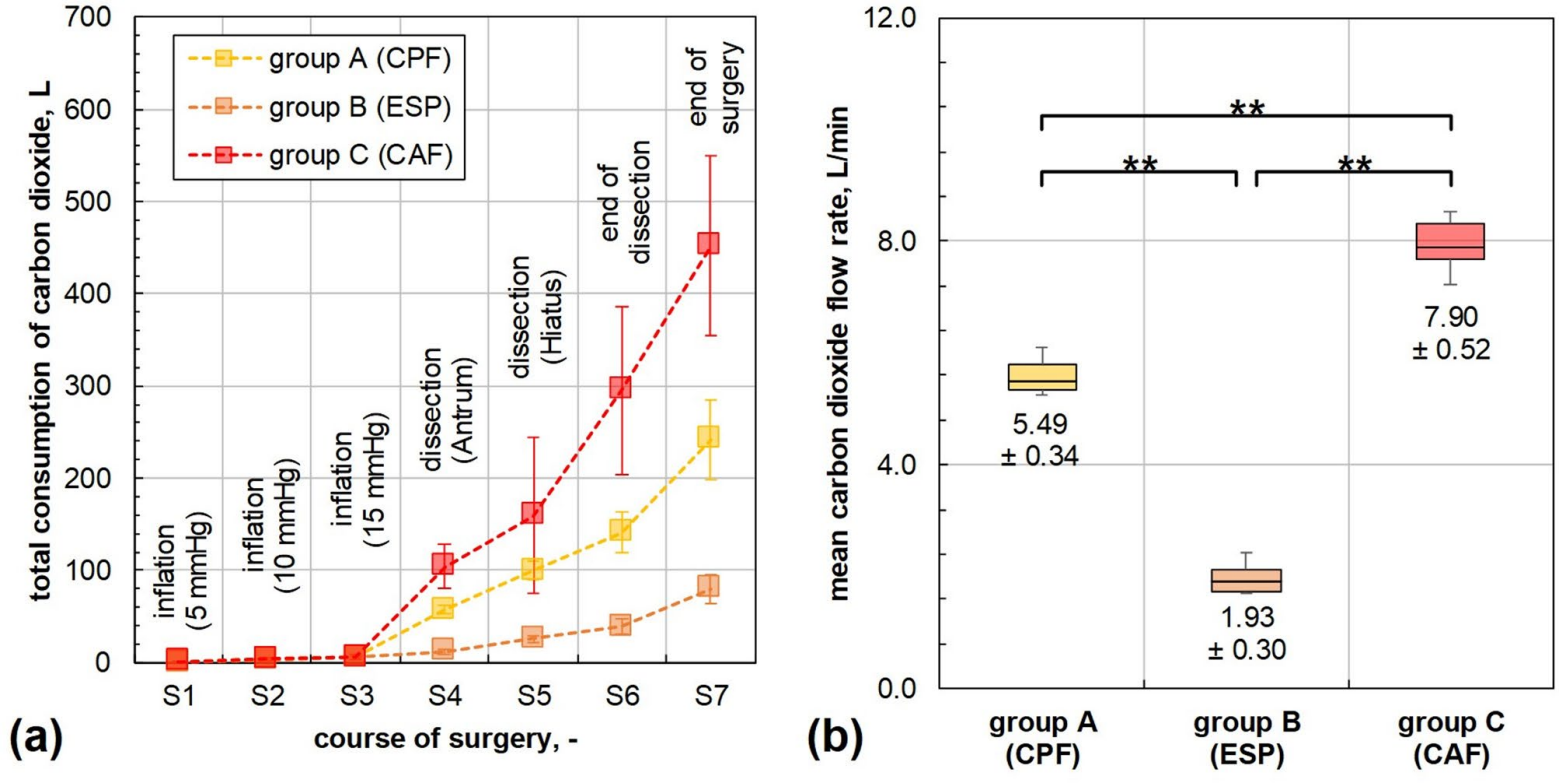
Cumulative carbon dioxide consumption (**a**) and mean carbon dioxide flow rate (**b**). A = group A (continuous passive filtration); B = group B (continuous electrostatic smoke precipitation); C = group C (continuous active filtration). ** = p < 0.01.

During the entire surgical procedure, the median CO_2_ consumption (Fig. 3a) was (242 ± 39.0) L for group A, (80.1 ± 13.7) L for group B, and (452 ± 87.9) L for group C. The consumption in group C was significantly higher than in groups A and B (p_C/B_ < 0.001; p_C/A_ < 0.001). The same holds true between groups A and B (p_A/B_ < 0.001).

The mean CO_2_ flow rate (Fig. 3b) was (5.49 ± 0.34) L/min for group A, (1.93 ± 0.39) L/min for group B and (7.90 ± 0.52) L/min for group C. Differences between the groups were highly significant (p_C/B_ < 0.001, p_C/A_ < 0.001, p_B/A_ < 0.001). Since group B technology does not rely on evacuation, the observed flow rate originates from unintentional leakage and the sample flow rate of (0.19 ± 0.04) L/min for aerosol characterisation.

The intraoperative capnoperitoneal pressure (Fig. S2b) was (14.3 ± 0.8) mmHg for group A, (15.8 ± 0.7) mmHg for group B and (15.2 ± 0.6) mmHg for group C (p_A/B_ < 0.01; p_A/C_ < 0.05; p_B/C_ < 0.01). Temporal fluctuations (Fig. S2a) of the intraoperative capnoperitoneal pressure are an indicator for the capnoperitoneal stability and were evaluated via the coefficient of variation. The overall coefficient of variation of the capnoperitoneal pressure was 5.43 % for group A, 4.12 % for group B and 4.23 % for group C (p_A/B_ < 0.01; p_A/C_ < 0.05; p_B/C_ = 0.05).

### Half-life of particle number concentration to assess surgical smoke elimination

**Fig. 4:**
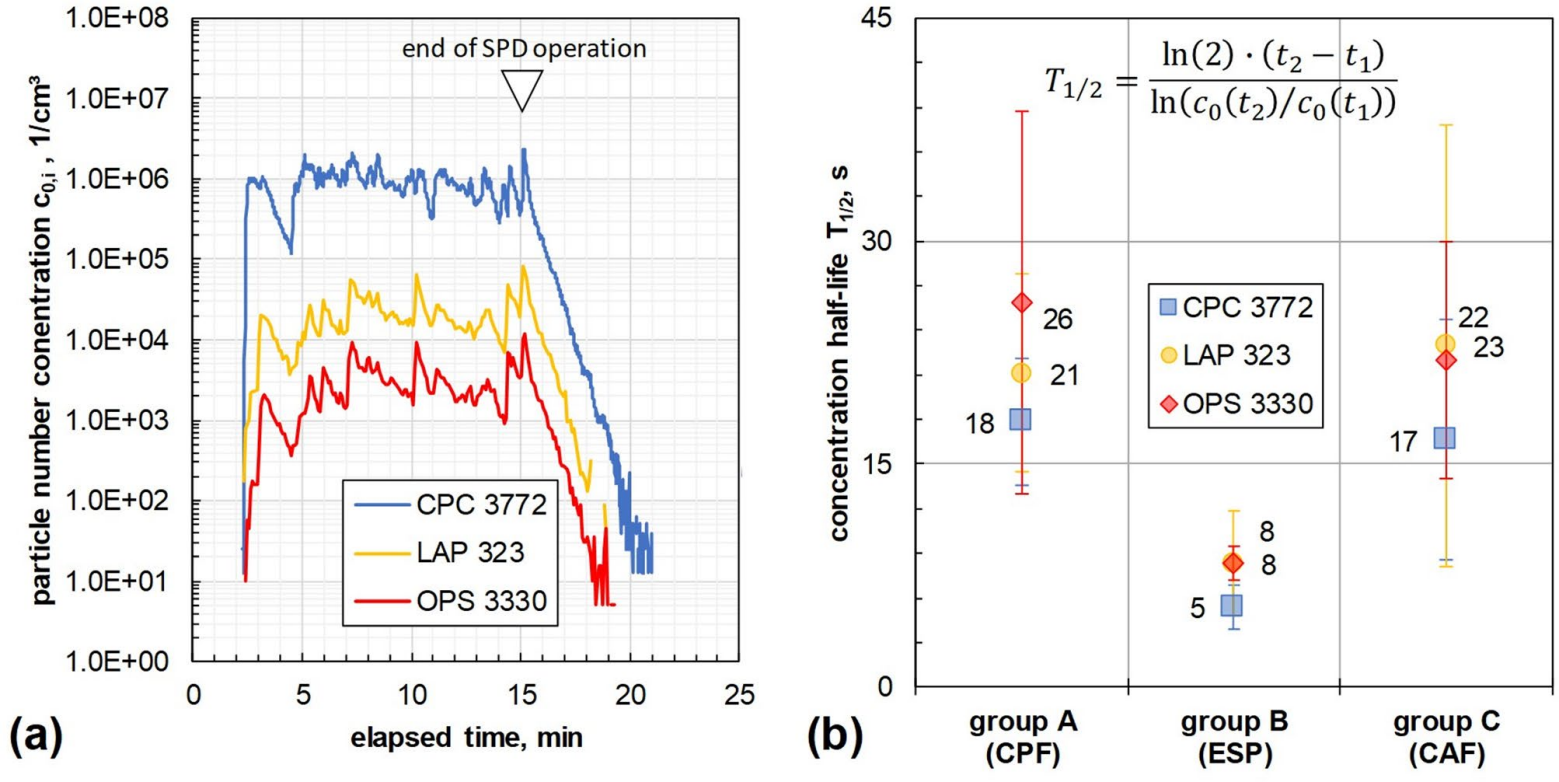
Example of the monitored intraperitoneal particle number concentration (**a**) and deduced concentration half-life values for each aerosol-analytical instrument (**b**). *CPC* condensation particle counter, *LAP* laser aerosol particle size spectrometer, *OPS* optical particle sizer, *CPF* continuous passive filtration, *ESP* electrostatic precipitation, *CAF* continuous active filtration.

Figure 4a shows the measured intraperitoneal particle number concentration over time for one trial of study group A. To assess the smoke removal efficiency, the concentration half-life T_1/2_ was calculated (see Fig. 4b) according to previous work^24^ from the exponential concentration decrease after use of SPD.

The lowest median concentration half-life over all instruments of T_1/2_(B) = (7.2 ± 2.6) s and thus the highest smoke removal efficiency was determined for study group B. Significantly higher (i.e., p_B/A_(T_1/2_) = 0.00003, p_B/C_(T_1/2_) = 0.00032) median concentration half-life values were determined for group A with T_1/2_(A) = (20.6 ± 8.6) s and group C with T_1/2_(C) = (18.3 ± 9.8) s. No significant difference in the median concentration half-life was be observed between group A and C (p_A/C_(T_1/2_) = 0.36).

### Quality of intraoperative visibility and intraoperative camera lens cleaning

In general, the intraoperative visibility was rated between “excellent” and “good” over the whole intervention for each study group. According to Fig. S3, the quality of visibility tended to be rated lower for group A (1.4 ± 0.5) than for group B (1.2 ± 0.4) and group C (1.3 ± 0.5).

The lowest total number of camera cleaning procedures per surgical intervention was determined for group C (0.2 ± 0.4), followed by group A (1.2 ± 0.8) and group B (2.4 ± 1.3). During dissection and stapler phase no cleaning was necessary for groups A and C, while group B required 0.4 ± 0.5 procedures (see Fig. S4). In summary, no significant differences between the study groups were observed with regard to the number of camera cleaning procedures (i.e., p-values > 0.05).

## Discussion

In view of preclinical data^23,24^ this clinical trial was conducted to evaluate the performance of different laparoscopic smoke management technologies. On the example of laparoscopic gastric sleeve, three different technologies were studied: continuous passive filtration (group A), electrostatic smoke precipitation (group B) and continuous active filtration (group C). Performance was evaluated based on particle concentration half-life (T_1/2_), capnoperitoneal stability, carbon dioxide consumption and intraoperative visibility.

The performance of each technology was evaluated based on several key metrics, including capnoperitoneal stability, CO_2_ consumption, smoke particle removal, and intraoperative visibility. A stable capnoperitoneum is imperative for ensuring the safety of laparoscopic surgery. Currently, pressure insufflators are categorized as either pulsatile or steady CO_2_ supply systems. The development of steady insufflators has been driven by the need to minimize fluctuations in capnoperitoneal pressure, which can result in unintended movements of the abdominal wall, trocar, and surgical site during procedures^24^. The trade-off of this is a dramatic increase in CO_2_ flow rate and consumption. In this study, a pulsatile insufflator was used for groups A and B, while a steady insufflator was employed for group C. Significant differences were observed between the study groups regarding both absolute capnoperitoneal pressure (target of 15 mmHg) and fluctuations. Group A exhibited the lowest capnoperitoneal pressure at 14.3 mmHg, followed by group C at 15.2 mmHg, and group B at 15.83 mmHg. Conversely, group B demonstrated the lowest fluctuations in capnoperitoneal pressure at 4.12 %, with group C at 4.23 % and group A at 5.43 %. Despite these differences, the overall deviations between group values were minimal, indicating that capnoperitoneal stability was satisfactory across all technologies.

In the context of laparoscopic surgery, the constant supply of CO_2_ is of paramount importance due to the inherent losses through the trocars^36^ and to optimize intraoperative visibility by effectively removing surgical smoke, particularly with evacuation-based technologies (groups A and C). The total CO_2_ consumption was found to be lowest in group B (80 L), followed by group A (242 L) and group C (452 L). When the duration of each laparoscopic intervention is considered, the CO_2_ consumptions can be expressed as flow rates, with group B showing the lowest flow rate at 1.9 L/min, compared to 5.5 L/min for group A and 7.9 L/min for group C. Since group B’s smoke removal does not rely on CO_2_ evacuation, the observed flow rate primarily reflects unintended leakage. From an economic and environmental perspective, the significantly lower CO_2_ requirement for surgical smoke removal via electrostatic precipitation (group B) provides a clear and relevant differentiating factor compared to evacuation-based technologies (groups A and C). However, the reduced CO_2_ flow rate of group B affected the number of camera cleaning events at the beginning of surgery. Group B experienced slightly more cleaning events than groups A and C, because the camera was not preheated before surgery. The low CO₂ flow rate caused thus an insufficient vapor removal from the abdominal cavity. Consequently, fogging due to condensation occurred early in the procedure. This effect resolved as the surgery progressed, with no significant difference in the total number of camera cleaning interventions observed among the three groups by the end of the operation.

In order to illustrate the relevance of our findings, a simple global approximation will suffice. Assuming that each of the 15 million laparoscopic interventions reported for 2018^37^ takes at least one hour, the data from this study suggest that replacing evacuation-based surgical smoke management technologies (groups A and C) with electrostatic precipitation (group B) could result in annual savings between 6,000 and 10,000 tons of medical CO_2_. Furthermore, given that the production of 1.0 L of medical CO_2_ results in the emission of 0.44 L of non-medical CO_2_, the total CO_2_ savings could range from 9,000 to 15,000 tons per year. These data are highly relevant and it is reasonable to assume that the actual CO_2_ savings are likely to be even higher, given the significant increase in laparoscopic surgery in recent years. The heavy dependence of the healthcare system on the supply of medical CO_2_ was particularly evident during the COVID-19 crisis - an adequate supply of medical CO_2_ is a significant risk factor for the safety of surgical patient care. In anticipation of shortages in the supply of medical CO_2_ during the COVID-19 crisis, some authorities developed contingency plans to ensure the supply of medical CO_2_ to the healthcare system^38^.

The half-life T_1/2_ (i.e., the time required to reduce the concentration of smoke particles by half) is an appropriate parameter for determining the effectiveness of laparoscopic smoke management technologies in preventing surgical smoke from entering the operating room atmosphere^23,24^. The current data confirm the results of the preclinical study, namely that electrostatic precipitation in group B is the most efficient laparoscopic smoke management technology as measured by T_1/2_ values. With average CO_2_ flows of 5.49 ± 0.34 L/min and 7.9 ± 0.52 L/min, respectively, it is approximately three times more efficient than continuous active and passive filter technology in groups A and C.

A potential limitation of this study is the lack of direct comparability between the groups in terms of body mass index (BMI) and gender distribution, which may have influenced the results. Group C was predominantly male, a population known to have more intra-abdominal visceral adipose tissue than females^39,40^. This may suggest that the higher BMI in group C may have adversely affected capnoperitoneal pressure stability and intraoperative visibility due to increased smoke particle release from greater visceral fat. However, as the study aimed to evaluate the efficacy of different smoke management techniques and T_1/2_ is independent of BMI and visceral fat, the results remain valid and are of clinically relevance.

During laparoscopic surgery, CO_2_ leaks of varying degrees, intentional or unintentional, occur constantly in the operating room, where the smoke poses a risk to health care workers^41^. However, the rate of smoke particle release leaks into the capnoperitoneum with electrostatic precipitation is significantly lower, by several orders of magnitude, compared to filter-based technologies. This is because a significant portion of the particulate matter released into the capnoperitoneum at the surgical site is rapidly deposited by electrostatic precipitation on the peritoneum or surrounding tissues. Electrostatic precipitation is therefore less likely than filter-based techniques to contaminate the operating room through accidental surgical smoke leakage ^24^.

It is also important to note that the effectiveness of electrostatic separation depends on the distance between the surgical site and the tip of the ion-emitting electrode. The intensity of the Gaussian field decreases as the cube of the distance increases, meaning that the effectiveness can decrease significantly as the distance from the surgical site to the electrode increases. Therefore, the electrode should be repositioned within the abdominal wall if the surgical field changes. However, if the electrode is placed too close to the surgical site, short circuits may occur if the electrode tip accidentally comes into contact with instruments or tissue. This will result in brief interruptions in smoke evacuation.

In summary, the study approved the main outcomes of prior performed ex-vivo^23^ and large animal in-vivo^24^ laparoscopic studies on the performance of different surgical smoke management technologies. The small sample size of this study was sufficient to evaluate the particle removal efficacy for each smoke management technology, but secondary outcomes like the impact on the operating time or on the number of camera cleaning procedures could not conclusively be addressed. Further studies are required, to examine result transferability from laparoscopic surgery to open surgery.

## Conclusion

All three techniques effectively eliminate smoke during laparoscopic gastric sleeve surgery while maintaining excellent visibility. Electrostatic smoke removal is the most effective method for managing smoke during the procedure, maintaining a stable capnoperitoneal pressure without extra CO_2_ consumption.

## Supplementary Information

Supplementary Information.

## Data Availability

Measurement data of the study are available from the corresponding author upon reasonable request.

## Abbreviations

BMI: Body mass index
CAF: Continuous active filtration
COVID-19: Coronavirus disease 2019
CPC: Condensation particle counter
CPF: Continuous passive filtration
DDS: Dynamic dilution system
EAD: Electrical aerosol detector
ESP: Electrostatic smoke precipitation
FCS: Field calibration system
HEPA: High efficiency particulate air (filter)
LAP: Laser aerosol particle size spectrometer
MFM: Mass flow meter
OPS: Optical particle sizer
SARS-CoV-2: Severe acute respiratory syndrome coronavirus type 2
SPD: Surgical power device
