# Supplementary material for: Evaluating the efficacy of smoke management technologies in laparoscopic sleeve gastrectomy: insights from a prospective, single-centre comparative study
Source: Sci Rep. 2026 Mar 23;16:9722. doi: 10.1038/s41598-026-43227-y (PMC13013633; doi:10.1038/s41598-026-43227-y)
Supplement: Supplementary file 1 — Supplementary Information. [file 41598_2026_43227_MOESM1_ESM.doc]

# Supplementary Information

**Figure S.1:** Capnoperitoneal volume at 15 mmHg. Legend: CPF = continuous passive filtration, CAF = continuous active filtration, ESP = electrostatic precipitation.

|  |  |
| --- | --- |

**Figure S.2:** Example of temporal evolution of capnoperitoneal pressure (a) and median capnoperitoneal values +/- standard deviation; Legend: CPF = continuous passive filtration, CAF = continuous active filtration, ESP = electrostatic precipitation.

**Figure S.3:** Quality of intraoperative visibility by Likert scale rating (1 = very good, 2 = good, 3 = fair, 4 = poor, 5 = very poor) at eight operative stages (S1 = prior to commencing dissection, S2 = upon entering the greater omental bursa, S3 = end of prepyloric dissection, S4 = during dissection towards the His angle, S5 = transection of the short gastric vessels; S6 = dissection of the fat pad at the esophageal hiatus, S7 = end of dissection, S8 = during stapled gastric transection). Legend: CPF = continuous passive filtration, CAF = continuous active filtration, ESP = electrostatic precipitation.

**Figure S.4:** Quality of intraoperative view by counting of camera cleaning procedures. Legend: CPF = continuous passive filtration, CAF = continuous active filtration, ESP = electrostatic precipitation.

**Estimation of non-medical CO2 emissions during production of medical CO2**

According to manufacturer information (<https://mosengg.com/co2-production-plant.html>, 30.03.2025), the production of 50 kg of technical CO2 requires 17 L of diesel and 35 kWh electric power for liquefying. According to (<https://www.umweltbundesamt.de/publikationen/co2-emissionsfaktoren-fuer-fossile-brennstoffe-0>, 30.03.2025), 3.17 kg of CO2 are emitted during combustion of 1,0 L diesel and 0,42 kg of CO2 are emitted for the production of 1 kWh electric power.
